# CULTURAL CONTEXTS AND BEYOND: THE NEED FOR VALUE SENSITIVE DESIGN IN TECHNOLOGIES TO SUPPORT CARE

**DOI:** 10.1093/geroni/igae098.0696

**Published:** 2024-12-31

**Authors:** Clara Berridge

**Affiliations:** University of Washington, Seattle, Washington, United States

## Abstract

It has long been understood in fields such as science and technology studies that technologies and data are not value-neutral, but digital technology interventions to support care partners of older adults are often treated as such. Understanding that values are cultural commitments that are not universally prioritized is critical for effective and non-harmful interventions. This presentation will describe ways in which values, assumptions, and priorities are embedded in digital interventions, drawing on findings from multiple studies of families’ experiences with care technologies. The importance of attention to norms, values, and expectations in care relationships, as well as contextual factors such as trauma history and intergenerational family cultural conflict will be discussed. Practical questions will be offered to guide the broader application of Value Sensitive Design in technology design and research to make all stakeholders’ values explicit, understand value tensions, and center and operationalize the values and needs of intended users.

